# A qualitative assessment in acceptability and barriers to use pre-exposure prophylaxis (PrEP) among men who have sex with men: implications for service delivery in Vietnam

**DOI:** 10.1186/s12879-021-06178-5

**Published:** 2021-05-25

**Authors:** Long Hoang Nguyen, Huong Lan Thi Nguyen, Bach Xuan Tran, Mattias Larsson, Luis E. C. Rocha, Anna Thorson, Susanne Strömdahl

**Affiliations:** 1 Department of Global Public Health, Karolinska Institutet, Stockholm, Sweden; 2 VNU University of Medicine and Pharmacy, Vietnam National University, Hanoi, Vietnam; 3 Institute for Global Health Innovations, Duy Tan University, Da Nang, Vietnam; 4 Faculty of Nursing, Duy Tan University, Da Nang, Vietnam; 5 Department of Health Economics, Institute for Preventive Medicine and Public Health, Hanoi Medical University, Hanoi, Vietnam; 6 Department of Health, Behaviours, and Society, Johns Hopkins Bloomberg School of Public Health, Baltimore, Maryland United States of America; 7 Department of Economics & Department of Physics and Astronomy, Ghent University, Ghent, Belgium; 8 Department of Medical Sciences, Section of Infectious Diseases, Uppsala University, Uppsala, Sweden

**Keywords:** Qualitative, Pre-exposure prophylaxis, PrEP, HIV, MSM, Vietnam

## Abstract

**Background:**

HIV Pre-exposure prophylaxis (PrEP) is being considered for implementation among MSM nationwide in Vietnam. However, there may be concerns about potential obstacles for PrEP adherence among Vietnamese MSM. This study aims to assess the acceptability to use PrEP, potential barriers and facilitators, and the preferences for PrEP service accessibility and delivery among Vietnamese MSM.

**Methods:**

Four focus group discussions (FGDs) were conducted with 30 HIV-negative MSM in January 2018 in Hanoi, Vietnam. FGDs explored MSM’s awareness regarding PrEP, perceived benefits and barriers of PrEP use, and willingness to use PrEP. FGDs were audio-recorded and transcribed verbatim. Content analysis was used.

**Results:**

The mean age of participants was 23.9 years old. Most participants realized the advantages of PrEP given its efficacy in HIV risk reduction and expressed high motivation and interest to use PrEP in the future. PrEP was considered as a supplement alongside condoms. Common concerns about PrEP included side-effects, forgetting to take the pill daily, stigmatization due to using PrEP, negative attitudes toward PrEP from other MSM and accessibility of PrEP. Participants would prefer an injectable PrEP regime if available as it was seen as easier to adhere to. Concerns were also raised that PrEP usage could be interpreted as an indication of engaging in sexual risk behavior for HIV, potentially causing suspicion in love relationships or by family and friends. Participants preferred to receive PrEP in civil business organizations and MSM-friendly clinics, and recommended that pharmacy stores would not be suitable for PrEP implementation due to lack of trust and fear of fake drugs.

**Conclusion:**

This study indicated a high level of willingness to use PrEP among MSM in Vietnam in combination with condom. Strategies to raise awareness of PrEP, reduce stigma towards PrEP, and improve the accessibility among MSM in Vietnam is needed. Existing MSM-friendly clinics were recommended to implement PrEP programs in Vietnam.

## Introduction

The burden of HIV/AIDS has been increasing in Vietnamese men who have sex with men (MSM) despite a decline achieved in other key populations namely people who inject drugs (PWIDs) and female sex workers (FSW) [1, 2]. Recent estimates from two National HIV Sentinel Surveillance Surveys (HSS) indicated that the prevalence of HIV in this population was 5.2% in 2015, 7.4% in 2016, and 10.8% in 2018 nationally, and 13% in Ho Chi Minh city – the biggest metropolitan area in Vietnam [3, 4]. The high frequency of condomless sex, multiple sex partners, substance use, as well as stigmatization and homophobia have been well-recognized as primary drivers for the spread of the HIV epidemic in this population [5–8]. However, poor access to and use of HIV prevention and testing services among MSM are reported even though many intervention programs have been implemented [9–12]. A new HIV prevention strategy is thus needed to reduce the burden of the HIV epidemic in this particular population.

Antiretroviral pre-exposure prophylaxis (PrEP) has been implemented among Vietnamese MSM [13, 14]. PrEP is highly efficacious in preventing HIV infection among MSM [15]. Data from previous observational and clinical studies in various countries revealed that PrEP was effective against HIV/AIDS transmission regardless of gender or sexual identity with acceptable safety [16–18]. The most recent population-level evidence from Australia indicates that PrEP implementation was associated with a 35% decline in new HIV cases among MSM [19]. Nonetheless, this strategy requires an at-risk HIV-seronegative MSM to take oral emtricitabine/tenofovir (FTC/TDF) daily, at least four times per week, or to follow the 2-1-1 on-demand regimen to be protected from HIV infection [15, 16, 20, 21]. In addition to oral pills, innovative strategies such as bi-monthly long-acting injectable PrEP (e.g., cabotegravir and rilpivirine) and rectal microbicide gel have been proven in recent years [22–24]. Other potential long-active PrEP candidates included a once-monthly oral pill from Merck, bi-annually long-acting injectable PrEP from Gilead, and slow release/long-acting implants such as Islatravir [25]. Long-active PrEP would be beneficial to ease adherence, while a microbicide gel could be used when having sexual intercourse in combination with a condom [14, 26, 27]. The proper use of PrEP is an important intervention to reduce the incidence of HIV/AIDS among MSM.

PrEP implementation is one of the key strategies to eliminate HIV/AIDS epidemic in Vietnam. In August 2020, the Prime Minister signed Decision No. 1246 / QD-TTg to approve the National Strategy aiming to end the AIDS epidemic by 2030, in which the expansion of HIV PrEP plays a key role [28]. Findings from an online survey in 2016 found high acceptance rates with different PrEP modalities among MSM in Vietnam with 65.8% preferring oral PrEP, while 69.8% and 85.4% showed interest in long-acting injectable PrEP and rectal microbicide gel, respectively [14]. Another study revealed that 76.1% of the MSM were willing to pay a median amount of 21.7 USD per month to use PrEP [29]. These data suggest that large scale implementation of PrEP among MSM in Vietnam is promising.

After two years of the implementation of the PrEP strategy, there were about 13,000 people in the general population utilizing PrEP [30]. Among those, more than 10,400 high-risk MSM accessed PrEP; however, this number is small when comparing to an estimated population of 122,000 – 512,000 MSM among the total population of Vietnam with approximately 99 million people [31]. Vietnam aims to reach 30% of entire MSM population accessing PrEP in 2025 and 40% in 2030 [30]. Effective PrEP delivery models for MSM has been challenging and there is a long way to reach the optimal number of people in the MSM target population. Successful execution requires more insights into MSM’s perceived barriers toward PrEP and their preferences for PrEP service delivery, which are vital to assure the benefits of this intervention to the target population [32]. Previous studies have used the quantitative approach to measure the willingness and barriers to use PrEP in MSM [14, 29], a study using qualitative methods can complement and provide in-depth insights to these issues, which are vital for developing further interventions to promote the PrEP use among MSM. We thus conducted this qualitative study aiming to explore the acceptability to use PrEP, identify potential barriers and facilitators, and preferences for PrEP delivery among Vietnamese MSM.

## Materials and method

### Study design and sampling method

From January to February 2018, we performed four focus group discussions (FGDs) in Hanoi, Vietnam with 30 individuals who were men, residing in Vietnam in the last 12 months, self-identifying as man who has sex with man (MSM), and having HIV-seronegative or unknown HIV status. Our sample varied in terms of socio-demographic (age, education, and occupation) and sexual identity characteristics. Table 1 shows the demographics of participants.

**Table 1:** Demographic characteristics of participants

Demographic characteristics	n	%
Age group (years)		
18-22	12	40.0
23-30	16	53.3
> 30	2	6.7
Income (VND^a^)		
< 3,000,000	8	26.6
3,000,000 - < 5,000,000	11	36.7
5,000,000 - < 10,000,000	11	36.7
Education		
Primary education	3	10.0
Secondary education	12	40.0
Tertiary education	15	50.0
Occupation		
Student	7	23.3
Self-employed	5	16.7
Business	5	16.7
Office worker	7	23.3
Artist	4	13.3
Other	2	6.7
Ever having HIV testing		
Yes	26	86.7
No	4	13.3
HIV status		
Negative	26	86.7
Unknown	4	13.3

^a^Conversion rate: 1 USD = 21,000 VND (2018 exchange rate)

We used purposeful sampling technique to recruit participants. Initially, we contacted the Vietnamese Men Who Have Sex with Men and Transgender Network and other Civil Business Organizations – that support lesbian, gay, bisexual, transgender (LGBT) advocacy in Vietnam - for detecting potential eligible MSM, introducing the study and asking them to participate in the study. MSM who showed interest in the study were contacted and once again informed of the study and invited to participate in a FGD at private room at the Hanoi Medical University. Tea and snacks were provided during the FGD. All participants provided verbal or written informed consent. This study was granted by the Institutional Review Board of Hanoi Medical University (Number 0618/HDDDDHYHN).

### Focus group discussions

Thirty MSM participated in four FGDs (6-8 MSM per FGD). Each FGD lasted on average 120 (between 108 to 132) minutes. The FGDs were performed in Vietnamese by two trained researchers who have a Master degree in Public Health and experience working with MSM and people who live with HIV, as well as experience in qualitative research (LN, HLTN).

After introducing the study and principles of FGDs, emphasizing confidentiality and the possibility to leave at any given time without any consequences, the MSM were asked for their informed acceptance to participate in the research and then written, or clear verbal (in the case they did not want to disclose their identities by giving a signature), informed consents were obtained. Then, discussions were conducted using an FGD guide developed by the research team. The guide included a series of open questions and probes to evaluate participants’ awareness and knowledge of PrEP, perceived benefits of PrEP, perceived barriers regarding PrEP uptake, PrEP adherence, MSM’s love and social relationships, the potential application of PrEP in Vietnam and the willingness to use PrEP. A short description of PrEP was initially provided at the beginning of the discussion, including how it differs from post-exposure prophylaxis (PEP) that was already offered at HIV clinics. Participants were encouraged to express their thoughts and experiences, as well as interact with other participants to discuss and share their opinions. After the FGDs, they were provided a short paper-based survey including socio-demographic information and history of HIV testing uptake. Participants were given compensation and travel support of a value of ~5 USD (~100,000 VND).

### Data coding and analysis

The FGDs were audio-recorded with the permission of all participants. The content was then transcribed, translated and checked for consistency by two researchers (LHN, HLTN). Any discrepancy between the two versions of transcripts were discussed with a senior researcher (BT). Data saturation was evaluated and obtained if participants discussed consistently across the first three FGDs. Then we applied a directed content analysis approach to identify themes [33]. Two researchers (LHN, HLTN) initially reviewed the transcripts based on the preconceived questions that were used to design the guide of FGDs. Then we applied an inductive approach to identify other emerging themes in the FGDs [34]. Coded quotes were presented in following themes: 1) Awareness of PrEP; 2) Perceived benefits of PrEP; 3) Perceived barriers in using PrEP and solutions; 4) Willingness to use PrEP and preferences for PrEP access. Quotes to highlights these themes in this text were selected through discussion by the research team.

## Results

**Fig. 1:**
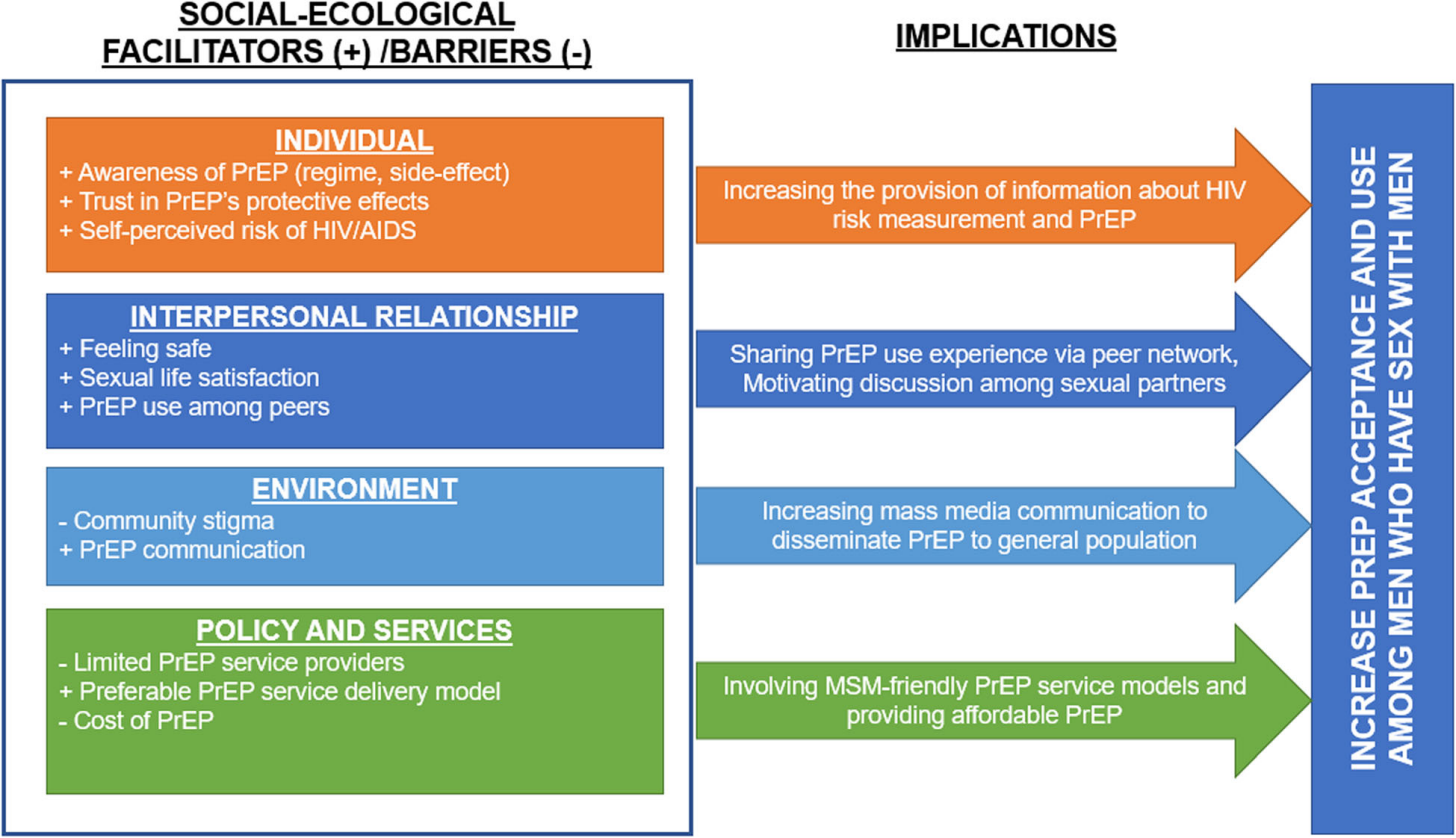
Framework to increase PrEP acceptance and use among men who have sex with men

In this section, we first present the awareness of PrEP among participants as well as their perceived benefits of PrEP in diminishing the risk of HIV infection (Theme 1). Then, we reveal the perceived barriers among participants to use PrEP and their suggestions on how to overcome these obstacles (Theme 2). Lastly, we depict the willingness of MSM to use PrEP and their preference to access PrEP in the future (Theme 3). Figure 1 presents a conceptual framework for facilitators and barriers in the acceptability and willingness to use PrEP among MSM.

### Theme 1: PrEP enables the feeling of protection and safety in sexual relationships

None of participants had used PrEP before the FDGs. Generally, knowledge about PrEP was low and only seven participants reported that they had heard about PrEP before the FGD. The majority of those who had heard about PrEP mentioned that they had discussed with friends on social network sites such as Facebook MSM groups or found PrEP information via online news on the Internet. One participant was aware of PrEP since his friend went abroad to get PrEP as he could not access PrEP in Vietnam. After having received information about PrEP as HIV prevention during the FGD session, all participants were in consensus that PrEP was a necessary preventive method for those frequently engaging in risk behaviors such as not using a condom, having multiple sex partners with unknown HIV or known HIV-positive status, group sex or when using substances during sex. Sex without a condom was deemed to be the major driver for the motivation to use PrEP among our participants. PrEP was deemed particularly helpful when a sex partner did not prefer to use condoms. With PrEP they would feel safe and thereby enhance their sexual satisfaction, which they also thought could improve their intimate relationships. Participants also expressed that condom less sex could give them more pleasure during anal intercourse. They thought that if they used PrEP, they could feel secure and become sexually liberated to try sexual behaviors they usually avoided because of fear of HIV infection.

*“ … I think if I use PrEP, I can tell my partner that I am safe [on PrEP]. I can ask if I do not need to use condoms. Actually, I don’t like using condom very much … ” (A03, FGD4)*

Most of the participants who had heard about PrEP were aware that PrEP could not prevent sexually transmitted infections (STIs). However, one participant viewed that infecting by STIs was acceptable because *“ … STIs are curable, unlike HIV, it’s another story. … .” (A05, FGD3)*; hence, this man was willing to trade-off his risk to STIs when using PrEP to be more satisfied with his sexual life. Nonetheless, in general, participants, both those knowing PrEP before the FGDs and those learning about PrEP during the FDGs, anticipated that PrEP should be considered an additional protective “layer” in combination with condoms rather than be treated as a complete prevention alternative. They perceived that *“ … The potential benefit is HIV prevention only …* ” *(A04, FGD3)* and “ *… It cannot prevent other diseases …* ” *(A02, FGD1)*. PrEP should be treated as *“ … a second guard alongside condom …* ” *(A03, FGD1)*, while condoms can be used to avoid both HIV and STIs.

### Theme 2: regime, side-effects, stigma, perceived low risk of HIV and cost hinder the acceptance of MSM to PrEP

Perceived barriers to PrEP use were discussed thoroughly among participants. Regimes and side-effects of PrEP were a major concern, followed by fear of stigmatization of persons using PrEP, sexual behaviors, lack of information, and accessibility.

#### Side-effects and Regime

Side effects of PrEP might be potential barriers for participants to start using PrEP, as these exists and no one can guarantee that you can “ … *take the medication in a period of time without any health consequences?” (A06, FGD2).* Another participant worried about the interaction between PrEP and other drugs. One participant who was a transgender person was aware that he could not use PrEP because he was currently using medications for transition. Several participants were afraid of the side-effects of PrEP because they had heard about side-effects from friends currently using antiretroviral therapy (ART) for HIV treatment. They believed that using PrEP could reduce their health and productivity significantly as ART had done for their friends.

*“ … I know a friend. He is HIV positive, and he is now on antiretroviral treatment. The drug reduces his health a lot. [He experienced] Vomit, pain, bad mood, lost weight. I am afraid when I use PrEP, I will be like him … ” (A06, FGD4)*

Participants acknowledged that PrEP had to be used consistently every day to achieve the optimal HIV preventive effect. Some participants believed that they could adhere to the daily regime by dividing PrEP into two packs: “ … *[One pack] At home and [one pack] at my office … ” (A03, FGD3).* Meanwhile, other participants reported that a daily regime could cause trouble and be problematic for them. The common reasons for not being able to adhere to the regime included 1) forgetting to take the pill and 2) if something unexpected happened in their lives, they would not be able to take the pill, such as work opportunity or going out (e.g. for work or entertainment). The importance of PrEP adherence was seen as the primary barrier of taking PrEP as PrEPs effectiveness was dependent on adherence.

*“ … You should take at an exact time every day, you know. You forget, and you will lose everything … ” (A04, FGD3)*

One participant suggested that the long-acting injectable PrEP should be provided along with oral medication. It would be beneficial for the PrEP users as they did not need to remember taking the pill daily, which could increase their uptake.

*“ … I read that we can inject PrEP, so we do not need to take PrEP orally anymore, and so we do not forget. I think it is a very good way. But I know there is only oral medication at that time … ” (A05, FGD4)*

#### Stigmatization

Stigma was considered as a considerable barrier that had to be overcome to use PrEP. The majority of participants reported that if they used PrEP, they could be judged and stigmatized by their friends, families and workmates. They expressed that PrEP could be seen as an indicator that they were high-risk individuals who engaged in sexual risk behaviors. While several participants positively perceive the benefits of PrEP as improving their sexual lives, some other participants believed that using PrEP could lead to mistrust in love or sexual relationships, which was viewed as problematic.

*“ … If I know that my partner uses PrEP, I will be suspicious of the problem, because this is for people at high risk. If he is not at risk, why would he take drugs?” (A06, FGD3)*

Therefore, participants suggested that information about PrEP as an HIV prevention for everyone should be disseminated widely to avoid this kind of stigma and to change the general population’s perceptions. This action would be helpful to create an informed and positive perception of PrEP and PrEP users.

*“ … Let people know more about PrEP. I think it’s essential to change their attitudes. If they know, they will not judge PrEP users because PrEP is concerned as a common HIV prevention method so that it’s for everyone … ” (A03, FGD1)*

One participant suggested that if people around him used PrEP, he would be willing to use PrEP too because he did not fear being stigmatized. Another participant told that to avoid stigma, he told everyone that he took vitamin supplements instead of PrEP. He also suggested that the label of drug box should not directly mention PrEP but replace to other names, which could help to reduce other people’s attention.

#### Perceived Low Risk Sexual Behaviors

Several participants who consistently used condoms or had only one sex partner expressed that they did not have any risk of HIV infection and therefore did not need to use PrEP. Participants suggested that MSM who did not engage in sexual risk behavior would not need or not be interested in PrEP.

*“I think people who are male sex workers, i.e., those at higher risk, may find it useful. For me, I always use a condom. I think condoms are good enough … ” (A07, FGD2)*

In addition, several participants expressed concerns that PrEP could lead the user to engage in more sex without condom (or unsafe sexual activities), which they feared could cause an increase in STIs. “ … *it is easy to expose STIs, they (i.e., PrEP users - author) will lose the use of condoms … ” (A03, FGD1)*

*“ … I am only worried about the opposite effect of it [PrEP-author]. Because you are safe, you don’t have risks of HIV infection, so you will have unprotected sexual intercourse more frequently … ” (A02, FGD2)*

#### PrEP Cost

The cost of PrEP was a concern expressed by three participants. A high cost would decrease users’ willingness to pay and adhere, and eventually affect the accessibility of PrEP.

*“I have heard that the price of this drug is 300,000 VND per month, so I am not sure that people will pay for it. Unless people have a lot of sexual relationships, they will be not willing to pay … ” (A04, FGD2)*

#### Attitude towards PrEP

Some participants were concerned that although PrEP is effective, condoms were favorable because its presence was visible, and they felt confident and secure by seeing and using a condom. While, for them, the effect of PrEP seemed uncertain and invisible, they could not fully believe in this medication.

*“ … I can use a condom and see how it works directly with my eyes. It makes me believe … While I cannot ensure how PrEP works … ” (A06, FGD4)*

### Theme 3: MSM-friendly civil business organizations and state clinics are preferable for PrEP delivery, but not pharmacy stores

Because PrEP was, at the time of this study, only implemented on a small pilot study scale in Hanoi, it was difficult for participants to know where they could access and buy PrEP. Participants suggested large-scale implementation of the PrEP program. The PrEP prescription refills could be freely offered through MSM Civil Business Organizations (CBOs) due to its close relationship with the MSM community.

*“ … I like taking the drug in some CBOs like Hai Dang club or iGirl group or the Hanoi Medical University clinic. They are friendly and help me a lot. I think we can provide the drug as similar to how condoms are distributed. I mean, free of charge … ” (A04, FGD4)*

State clinics were also referred to as a safe trusted source. Pharmacy stores were the least preferable due to a lack of trust in pharmacies in handing out proper medication. One participant stated that *“ … I think PrEP can be provided in drug stores. But I am afraid of fake drugs. How can we know it is real PrEP?...” (A01, FGD2).*

Regarding procedures, one participant expressed his concern that if he had to undergo a physical examination and take tests if he wanted to use PrEP, this could reduce his interest in taking PrEP.

*“For me, I feel uncomfortable because I need to undergo many rigorous tests such as liver or kidney, so I need to be healthy if I want to take the drug.” (A03, FGD3)*

Overall, most of the participants expressed their willingness to use PrEP in the future if it was affordable. They also suggested that PrEP should be used not only by MSM but also be available to other people at risk of HIV. By universally providing PrEP to those at risk in the general population, they expected that the PrEP price would reduce significantly and become more affordable and “ … *everyone will be easier to access … ” (A03, FGD1).* However, some participants anticipated that PrEP would not be available and commonly used among MSM in the near future unless it was implemented in large scale programs for all at risk of HIV in Vietnam with clear information about effectiveness.

*“I think PrEP certainly has the future although it may take a long time. Because even condoms are just accepted by people recently, it is important to demonstrate the effectiveness of the drug, and its side effects” (A03, FGD4).*

## Discussion

High acceptance of PrEP among participants was found, which supports previous quantitative assessments [11, 14]. Most of the participants perceived PrEP as a promising intervention tool given its effectiveness in HIV transmission reduction and expressed their interest in using PrEP in the future. Participants viewed PrEP as a supplement alongside condoms and not as the primary HIV prevention alternative. The main perceived barriers for using PrEP included lack of PrEP knowledge, difficulties in adhering to a daily regime and fear of being viewed and stigmatized as someone who engages in high risk behaviors for HIV.

In Vietnam, PrEP remains a relatively new HIV prevention method compared to other strategies such as condom or test and treat programs [35]. Even though the first PrEP clinic in Ho Chi Minh city was opened in 2017, the awareness of PrEP in the general population and at-risk populations such as MSM is expected to be low. It is thus not surprising that most of the participants in our study had not heard of PrEP before the FGDs. Despite low awareness, our study identified a high level of willingness to use PrEP in the future. This finding could be explained by the fact that our participants had high level of education, which is a significant predictor of active health-seeking behavior [36]. High levels of education were also observed homogeneously in other study samples of Vietnamese MSM [5, 13, 37–41], indicating the feasibility in disseminating PrEP information among the MSM population across Vietnam’s provinces. In addition, the major source of PrEP information among participants was the Internet and particularly social media, which were consistent with a previous study [14]. These online information channels are also common platforms for finding sex partners as well as sexual health and HIV/STI information [40, 41]. Therefore, a variety of communication channels, particularly social media web sites, should be considered and piloted carefully for maximizing the number of MSM reached by information on future PrEP implementation programs.

Although PrEP could be used to alleviate the risk of HIV infection, most of MSM in our study did not consider using PrEP alone but preferred comprehensive PrEP programs including other safe sex interventions particularly condoms as also recommended by WHO [42–45]. Similar to our findings, a qualitative study among MSM in Malaysia reported that condom use provided a visible physical barrier by separating bodily fluids after sexual intercourses, which was deemed as insurance to feel safe during sex, and protected from HIV transmission [46]. The perception of the condom's visible presence is described as a critical factor for trusting the protective effect against HIV infection, while biomedical approaches posed scientific uncertainty which raised doubts about their effectiveness in preventing HIV transmission [47]. Moreover, PrEP is not effective in STIs prevention [48]; thus, although several participants recognized STIs as less serious threats, most of our participants were afraid of STI infections if they only used PrEP as prevention. Notably, prior studies revealed that PrEP users were more likely to have motivation for condomless sex, as well as reduce condom use during their sexual practices [49, 50]. Therefore, participants’ responses could be influenced by the social desirability bias, when participants felt pressured that they had to state the greater role of condom compared to PrEP before other participants in the FDGs. Therefore, the findings should be taken with caution. Comprehensive PrEP programs including safer sex counseling with a focus on condom use would create trust and increase uptake among MSM.

We focused FGDs on the oral PrEP because as it will likely be the first modality provided by the government in Vietnam at a larger scale in the coming years. Our study was conducted in 2018, when the existing PrEP regime at that time requires using daily for optimal effectiveness. However, currently, MSM could have many PrEP choices and the current regime requires taking the medication at least four times per week; thus, MSM do not need to achieve daily “perfect” adherence [15, 16, 20, 21]. PrEP side-effects and adherence problems were the predominantly hypothetical concerns of our participants, which could reduce the interest in PrEP as well as medication adherence [11, 37, 51]. Noticeably, the drug-drug interactions between PrEP and gender affirming hormone therapy for transgender people has been raised during the discussion. A previous review suggested that the minor interactions between PrEP and gender affirming hormone therapy should not be recognized as a barrier for PrEP uptake in this group [52]. Therefore, educational campaigns for MSM should be performed to illustrate that side-effects of PrEP (Emtricitabine-Tenofovir) were mild and transient for both cis and transgendered people based on the best available evidence, and how these improve after about a month of usage, which could be also beneficial in Vietnam [53]. Meanwhile, individual strategies to adhere to PrEP such as text message reminders via mobile phones, alarm clock and keeping the pills at hand both at home and work, should be part of counseling in PrEP programs to facilitate adherence [54, 55].

Social stigma and relationships were also crucial barriers to the willingness to use PrEP. These issues have also been observed in Malaysia and India [46, 56]. Stigmatization of people living with HIV in Vietnam is still substantial, and PrEP use can be interpreted as engaging in HIV-related risk behaviors or even misinterpreted as living with HIV [57]. This stigma can hinder individuals from accessing PrEP [55]. Communication interventions should be developed and implemented to educate people that PrEP is a preventive medication for everyone at HIV risk, not only MSM, FSW, PWIDs but also other at-risk populations [14, 46, 56]. The individual risk of HIV should guide PrEP use and prescription decision as recommended by WHO [42–45]. Moreover, manufacturers could consider designing the PrEP drug box to diversify it from HIV medication and avoid sensitive information.

In our study, service cost and providers were potential obstacles for PrEP use, a finding that is similar to prior studies [14, 46, 56]. The high cost of PrEP would result in intermittent PrEP use. Thus, a variety of PrEP packages or delivery options with different prices, or preferably free-of-charge, should be developed [14]. Peer outreach and CBOs have played an important role in delivering interventions to the MSM population in Vietnam [13]. Our participants preferred PrEP implementation through MSM-friendly CBOs. Based on their connections with the MSM populations, it is feasible to assign these CBOs as PrEP providers [46]. However, the main barrier is that well-trained medical professionals should also be allocated to these CBOs to ensure the quality of drug dispensed and medical follow-up. This criterion is strictly required in the current national strategy for PrEP program implementation [58]. We also found that our participants did not prefer pharmacy stores to provide PrEP because they feared “fake drugs” (i.e., counterfeit drugs). Several studies have pointed out that drug counterfeiting in Vietnam is an issue (e.g., anti-malaria [59, 60]). Another report of the EU-Vietnam Business Network indicated an increase of counterfeit drugs in the Vietnam pharmaceutical market [61]. Our data suggests that pharmacy stores would not be preferable for implementing PrEP programs for MSM due to trust issues.

This study has some limitations. First, during the study period, PrEP was not widely accessible in Vietnam and we thus asked participants about their willingness and barriers to a hypothetical scenario where PrEP would be available [46, 62, 63]. If PrEP were widely available, the perception of other facilitators and barriers could have emerged, particularly due to social influence among peers and daily experience with the regimen. Second, the generalizability of the findings is limited due to the qualitative design and purposeful sampling used. Our samples were relatively young, and recruited from different LGBT community organizations, whose members may be were more socially connected and more likely to have received sexual health education. Caution should be taken about applying the results to the wider MSM population as this is a qualitative study. However, as data saturation was reached, we believe that our findings could partly transfer to other similar settings with similar context to explore the social-cultural facilitators and barriers that can occur during the implementation of PrEP. Finally, the nature of FDGs, which requires the presence of participants during the discussion, might cause social desirability bias. Thus, they might conform to the group opinion instead of sharing their thoughts. We have attempted to generate a pleasant and friendly atmosphere to promote open discussions among participants to minimize this issue.

## Conclusion

This study found a high willingness to use PrEP among MSM in Vietnam. Strategies to raise awareness of and support positive perceptions about PrEP will be helpful to reduce stigma towards PrEP/ART and should be considered when implementing comprehensive PrEP programs in Vietnam. Online communication strategies were preferred among MSM. The preferred implementation modalities for MSM was through MSM-friendly clinics and MSM civil business organizations, while pharmacies were deemed not appropriate due to worry about counterfeit drugs.

## Data Availability

The data that support the findings of this study are available from the Hanoi Medical University but restrictions apply to the availability of these data, which were used under license for the current study, and so are not publicly available. Data are however available from the authors upon reasonable request and with permission of Hanoi Medical University (contact hoang.nguyen@ki.se).

## Abbreviations

AIDS: Acquired immunodeficiency syndrome
FGD: Focus group discussion
HIV: Human immunodeficiency virus
MSM: Men who have sex with men
SMS: Short message service
STIs: Sexually transmitted infections
CBOs: Civil Business Organizations
PrEP: Pre-exposure Prophylaxis
ART: Antiretroviral therapy
